# Exercise-induced mitochondrial protection in skeletal muscle of ovariectomized mice: A myogenic E_2_ synthesis-independent mechanism

**DOI:** 10.1016/j.redox.2025.103735

**Published:** 2025-06-21

**Authors:** Xu Tian, Yi Hu, Tao Li, Fangfang Yu, Tingting Li, Xiangyang Tian, Yiwei Feng, Qiuling Zhong, Yifan Meng, Wei Chen, Rengfei Shi

**Affiliations:** School of Exercise and Health, Shanghai University of Sport, Shanghai, China

**Keywords:** Muscle weakness, Myogenic 17β-estradiol, Mitochondrial function, Exercise training

## Abstract

**Background:**

Skeletal muscle, a 17β-estradiol (E_2_)-sensitive tissue, is prone to accelerated aging due to postmenopausal E_2_ deficiency and subsequent mitochondrial dysfunction. While exogenous E_2_ treatment has been shown to protect against mitochondrial damage in ovariectomized rodents, the impact of exercise-induced local E_2_ production in skeletal muscle on mitochondrial function remains to be determined. This study investigated exercise-mediated mitochondrial protection in ovariectomized mice and the contribution of myogenic E_2_.

**Methods:**

Female C57BL/6J mice (8-week-old) were divided into Sham, OVX, and OVX + ET groups (N = 12). OVX mice underwent bilateral ovariectomy, with the OVX + ET group performing 8 weeks of treadmill exercise starting 10 weeks post-surgery. Functional tests (grip strength, fatigue resistance) and gastrocnemius analyses (morphology, mitochondrial function, E2/antioxidant levels, and protein expression) were conducted. Parallel experiments in muscle-specific aromatase knockout (MS-ARO-CKO) mice included E2 supplementation via subdermal pellets.

**Results:**

18 weeks after ovariectomy (OVX), C57BL/6J mice exhibited significant reductions in grip strength (∼30 %), rotarod performance (∼57 %), and grid hanging performance (∼92 %). Concomitantly, OVX led to marked decreases in mitochondrial respiration (p < 0.05) and antioxidant capacity (p < 0.05) in the gastrocnemius muscle, accompanied by alterations in mitochondrial quality control and antioxidant signaling proteins (p < 0.05). Exercise intervention effectively attenuated these OVX-induced deficits, accompanied by a 66 % increase in E_2_ levels and upregulation of aromatase (ARO) activity and expression (p < 0.05). In MS-ARO-CKO mice model, exercise failed to improve the impaired antioxidant capacity induced by OVX. However, exercise, similar to estrogen supplementation, restored mitochondrial function and related protein expression abnormalities induced by OVX (p < 0.05).

**Conclusions:**

Our findings demonstrate that the protective effects of exercise on skeletal muscle mitochondria involve multiple mechanisms, independent myogenic E_2_ Synthesis, providing novel insights for improving skeletal muscle health in postmenopausal women.

## 1 Introduction

Aging, a process characterized by progressive tissue and organ dysfunction, manifests in skeletal muscle as a decline in both mass and strength, often culminating in sarcopenia and frailty. This muscle weakness is a significant contributor to falls and fractures, the second-leading cause of injury and mortality among the elderly [1]. In women, menopause characterized by ovarian failure marks a stage of aging associated with a shift in the systemic steroid hormone profile from a regularly fluctuating estrogen cycle to sustained low levels. This hormonal shift has been shown to adversely affect estrogen-responsive tissues, including skeletal muscle [2]. Premenopausal women generally exhibit superior muscle properties compared to postmenopausal women, and postmenopausal hormone replacement therapy (HRT) can mitigate or even reverse age-related muscle decline in the early postmenopausal years [[3], [4], [5]]. However, the benefits and risks of HRT remain controversial due to potential links with cardiovascular disease and breast cancer, which has limited its widespread adoption [6,7]. Therefore, it is crucial to investigate the role of estrogen in skeletal muscle and to explore safe and effective alternative options to maintain muscle health in postmenopausal women.

Mitochondria, the powerhouses of cells, are pivotal in maintaining skeletal muscle metabolic homeostasis and function. Through oxidative phosphorylation, mitochondria generate ATP and are involved in the production of reactive oxygen species (ROS), ion signaling, cell differentiation, growth, and apoptosis [8,9]. In skeletal muscle, mitochondria serve as a primary target for estrogen and its receptor-mediated signaling. Studies have demonstrated that estrogen deficiency induced by ovariectomy leads to decreased mitochondrial respiratory capacity, biogenesis, and ATP synthesis in skeletal muscle. However, these adverse effects can be reversed by 17β-estradiol (E_2_), the most biologically active estrogen, replacement therapy [10,11]. E_2_ has been shown to directly influence mitochondrial membrane fluidity and function, thereby protecting muscle cells from oxidative damage [11]. Moreover, E_2_ modulates mtDNA transcription and replication, as well as preserves mitochondrial morphology and function, through the activation of estrogen receptor (ERα) signaling pathways [12]. Despite the incomplete understanding of the molecular mechanisms governing E_2_ and its receptors in regulating skeletal muscle mitochondrial homeostasis, therapeutic interventions aimed at restoring mitochondrial function represent a promising avenue for combating muscle weakness associated with estrogen deficiency.

Increasing evidence supports estrogen synthesis and its actions can be tissue-specific, suggesting that this localized mechanism may serve to partially counterbalance the systemic estrogen deficiency resulting from the loss of ovarian function in postmenopausal women [13,14]. Analogous to tissues such as brain, adipose, and bone that are well-known for estrogen synthesis, skeletal muscle also expresses the enzyme aromatase cytochrome P450 (hereafter termed aromatase), catalyzing the aromatization of testosterone into estradiol [15]. While previous studies have primarily focused on the protective effects of exogenous E_2_ on skeletal muscle mitochondria in ovariectomized rodents, the potential role of muscle locally synthesized E_2_ in mitigating mitochondrial dysfunction remains underexplored. Interestingly, our prior research revealed that exercise training upregulated aromatase expression and elevated E_2_ levels in the skeletal muscle of ovariectomized rats [16]. Given the evidence for tissue-specific estrogen synthesis and the presence of aromatase in skeletal muscle, it is plausible that locally produced E_2_ could play a significant role in maintaining mitochondrial health in postmenopausal women.

In the present study, we investigated the impact of exercise, a well-established intervention for improving skeletal muscle health, on mitochondrial function in ovariectomized mice. Our hypothesis was that exercise could induce mitochondrial protection in skeletal muscle through a mechanism involving local E_2_ synthesis. This study aimed to elucidate the mechanisms underlying these exercise-induced mitochondrial benefits, particularly in the context of estrogen deficiency. We also sought to provide insights into potential therapeutic strategies for maintaining muscle health in postmenopausal women.

## 2 Materials and methods

### 2.1 Animals models

All animal experimental procedures were approved by the Animal Care and Use Committee of the Shanghai University of Sport (Ethics No. 102772022DW031). All research involving animal experimentation followed the National Institute of Health guidelines on the care and use of animals. *Ckmm*-Cre (SJ-006475) mice were purchased from the Shanghai Model Organisms Center (Shanghai, China). All male and female *ARO*^flox/flox^ mice were purchased from the GemPharmatec Company (Nanjing, China). *ARO*^KO^ mice (*ARO*^flox/flox^/*Ckmm*-Cre^+^) were generated by crossbreeding *ARO*^flox/flox^ and *Ckmm* promoter driven Cre transgenic mice. *ARO*^KO^ mice and their wild-type (WT) littermates were used in the experiments. All mice were housed at 22–25 °C in a 12 h light-dark cycle and fed adlibitum with standard mouse feed and water throughout the experiments.

### 2.2 Ovariectomy procedures

At 8 weeks of age, female mice were randomly assigned to either a sham surgery or bilateral ovariectomy group, following our established protocol [17]. Briefly, mice were anesthetized with isoflurane and secured on an operating table. For ovariectomy, a 1 cm dorsal incision was made to excise both ovaries; in contrast, the sham procedure involved the removal of an equivalent volume of fat tissue adjacent to the ovaries, followed by wound closure. Ovariectomy success was verified by the absence of estrous cells in daily vaginal smears over a ten-day period.

### 2.3 Estradiol replacement

After 10 weeks post-ovariectomy, mice in the OVX + E_2_ group were implanted with a subcutaneous E_2_-releasing pellet (SE-121, 0.36 mg, 60 days, IRA). The procedure involved anesthesia with isoflurane, lifting the dorsal skin, and creating a small incision to insert the pellet approximately 2 cm deep. The selected E_2_ dosage was based on prior findings to ensure serum levels remained within the physiological range [18].

### 2.4 Treadmill training protocol

Mice in the OVX + ET group commenced a one-week adaptive treadmill training at 17 weeks of age, followed by an 8-week (5 days/week, 60 min/day) moderate-intensity treadmill exercise intervention [19,20]. During the adaptive training phase, the treadmill was set at a speed of 10 m/min with a 0° incline. For the formal exercise intervention, the initial speed was set at 15 m/min for a constant-speed run for the first 15 min, after which the speed was incrementally increased by 1 m/min every 15 min, reaching 18 m/min by the 60th minute. The incline was maintained at 0° throughout the training. Sham and OVX groups were kept on a normal diet, with weight changes and food intake monitored for all mice.

### 2.5 Body composition measurement

Body composition analysis was performed on all mice using an Encho MRI body composition analyzer before and after the exercise intervention. Briefly, following the manufacturer's guidelines and calibration of the device, mice with known body weights were placed into the measurement chamber for testing, with lean mass and fat levels being recorded.

### 2.6 Grip strength test

The grip strength of mice was evaluated using a Grip Strength Meter (YLS-13A, Jinan Yiyan Technology Co., Ltd. Jinan, China) [18]. After the mouse grasped the rod with all limbs, the tester pulled the mouse horizontally backward at a constant speed by holding the tail, and the peak grip force displayed on the gauge was recorded. Seven consecutive readings were taken, excluding the highest and lowest values, and the average of the remaining five measurements was calculated and normalized to the mouse's body weight to determine the final grip strength value. Grip strength tests were conducted before and after the exercise intervention.

### 2.7 Rotating rod fatigue test

A rotating rod fatigue apparatus was utilized to assess the fatigue resistance of mice [21]. On the day before the formal test, mice underwent an adaptive exercise where they stayed on the rod rotating at 15 rpm for 5 min. During the formal test, after a 2-min pre-adaptation at 10 rpm, the rod speed was increased to 30 rpm for the actual test, and the time was recorded until the mouse fell off (if a mouse stayed on the rod for over 30 min, it was removed, and the time was recorded as 30 min). Two tests were conducted within a week with a 4-day interval between tests, and the average of the two tests was calculated and normalized to the mouse's body weight to determine the final fatigue resistance value.

### 2.8 Inverted grid test

The fatigue characteristics of the mice's limbs were tested using an inverted grid hanging test [21]. For the test, mice were placed on an inverted grid panel (40 cm × 80 cm; grid hole size 10 cm × 10 cm), and the time they could hang before falling was recorded (the maximum duration allowed for each trial was 6 min; if exceeded, the time was recorded as 6 min). Two tests were conducted within a week with a 4-day interval, and the average of the two tests was calculated and normalized to the mouse's body weight to determine the final fatigue resistance value from the inverted grid test.

### 2.9 Histological analysis

Gastrocnemius (GAS) muscles were carefully dissected, placed into a mold containing O.C.T. embedding medium (SAKURA), and rapidly frozen in isopentane cooled by liquid nitrogen for approximately 15 s. Samples were stored at −40 °C prior to cryosectioning. Tissue sections, 8-μm in thickness, were prepared and stained with hematoxylin and eosin (H&E), modified gomori trichrome (MGT), or NADH-tetrazolium reductase (NADH-TR). Observations were conducted using a brightfield microscope equipped with a CCD camera (BX-53, Olympus, Japan) for morphological and pathological assessment.

### 2.10 Immunostaining

For immunofluorescence, muscle sections were fixed in 4 % paraformaldehyde (PFA) for 10 min and then blocked with 10 % normal donkey serum for 1 h at room temperature. The sections were then incubated with appropriate primary antibodies overnight at 4 °C,including anti-aromatase (Invitrogen, catalog #SD2376082A, diluted 1:200), anti-estrogen receptor (anti-ER) (Invitrogen, catalog #RH238843, diluted 1:200), and anti-E_2_ (Abcam, catalog #131413, diluted 1:100). After washing with 0.1 % Triton X-100, sections were incubated with Alexa Fluor-tagged donkey anti-mouse/rabbit secondary antibodies (Thermo Fisher) for 1 h. Following three further washes, sections were mounted in Vectashield with DAPI (Vector Laboratories). Images were captured on an LSM-700 confocal microscope (Carl Zeiss) using 40–100 × Neofluor objectives at 1024 × 1024 pixels and analyzed with LSM-700 Meta software. Four to five sections per animal were assessed for representative immunofluorescence.

### 2.11 Mitochondrial morphology analysis

The alterations in the mitochondrial morphology of the GAS muscles were observed by transmission electron microscopy (TEM). Immediately after the mice were sacrificed, GAS muscles were rapidly excised and trimmed into 1 mm^3^ cubes. They were then fixed in 2.5 % glutaraldehyde, and postfixed with 1 % osmium tetroxide, dehydrated, and then embedded in epoxy resin. The embedded samples were polymerized by heating and subsequently sectioned into 60 nm ultrathin slices using an ultramicrotome. Sections stained with 2 % uranyl acetate and 2.6 % lead citrate under a HT7800 microscope (Hitachi, Japan) at 25,000 × magnification.

### 2.12 Mitochondrial respiration

Mitochondrial respiration in red GAS muscle fibers was evaluated following established protocols [11,18]. Freshly dissected muscle was treated with 52.5 mg/mL saponin in Buffer X (2.77 mM CaK_2_EGTA, 7.23 mM K2EGTA, 5.77 mM Na_2_ATP, 6.56 mM MgCl_2_·6H_2_O, 20 mM Taurine, 15 mM Na_2_Phosphocreatine, 20 mM Imidazole, 0.5 mM Dithiothreitol, 50 mM K-MES, pH = 7.1) and incubated at 4 °C, then washed and transferred to Buffer Z (0.5 mM EGTA, 3 mM MgCl_2_·6H_2_O, 60 mM K-lactobionate, 20 mM Taurine, 10 mM K_2_HPO_4_, 20 mM HEPES, 110 mM Sucrose, 1 g/L BSA, pH = 7.1) for 15 min. The high-resolution respirometry Oxygraph-2k (O2k, OROBOROS Innsbruck, Austria) was utilized to measure mitochondrial respiration, with the chamber hyperoxygenated to ∼450 mM. The assay commenced with the sequential addition of l-Malic acid (Mal; 1 mM), l-Glutamic acid (Glu; 10 mM), Adenosine 5′-diphosphate (ADP; 5 mM), Cytochrome C (Cyt-C; 10 μM), Succinate (Suc; 10 mM), Rotenone (Roc; 0.5 mM), Antimycin A (Ant; 2.5 mM), Ascorbate sodium salt (Asc; 2 mM), and TMPD (0.5 mM), with respiration rates normalized to the wet weight of permeabilized fibers.

### 2.13 Measurement of aromatase activity

Aromatase activity was quantified by assessing the conversion efficiency of testosterone to E_2_ in vitro [22,23]. GAS muscles were homogenized in a cold phosphate buffer solution (10 % Glycerol、10 mM NaH_2_PO_4_、40 mM Na_2_HPO_4_、1 mM EDTA、3 mM NaN_3_, 1 mM DTT; pH 7.4) using an Eppendorf homogenizer at 12,000 g for 20 min. Total protein content of the resultant supernatant was quantified using the bicinchoninic acid (BCA) protein assay. Test tubes were prepared with 100 μL muscle homogenate (1 mg/ml), 4 μL testosterone (2 μg/mL in propylene glycol), and 40 μL NADPH solution (3 mg/mL in amphibian saline). Following a 30-min incubation at 37 °C, reactions were halted by freezing. E_2_ levels were assessed using a commercially available enzyme-linked immunosorbent assay (ELISA) kit (Cloud-Clone, China), according to the provider's guidelines. Aromatase activity was calculated based on the quantity of E_2_ generated per milligram of skeletal muscle tissue protein in 1 h.

### 2.14 Biochemical analysis

GAS muscle tissues were homogenized in ice-chilled PBS, and the supernatant was collected as the test sample subsequent to centrifugation. Enzymatic activities of superoxide dismutase (SOD), catalase (CAT), and glutathione peroxidase (GPx), as well as malondialdehyde (MDA) content, were quantified using assay kits from Njjcbio (China). All procedures adhered to the manufacturer's instructions.

### 2.15 Measurement of ATP levels

Skeletal muscle ATP levels were determined using an Enhanced ATP Assay Kit (Beyotime, China), following the manufacturer's protocol. ATP content was expressed in arbitrary units, as depicted in the results.

### 2.16 Quantification of mtDNA

DNA was extracted from GAS muscles tissue using a Genomic DNA Extraction Kit (TAKARA, Japan). Quantitative PCR was conducted to quantify mtDNA with TB Green® Premix Ex Taq™ II (TAKARA, Japan) on a QuantStudio™ 6 Flex Fast Real-Time PCR System (Life Technologies). The copy number of mtDNA encoding cytochrome B (*Cytb*) was normalized to nuclear DNA encoding cytochrome *c* (*Cycs*). The following primers were used: Cytb (F: 5′- TGCATACGCCATTCTACG-3′ and R: 5′- ATGGGTGTTCTACTGGTTG-3′) and *Cycs* (F: 5′- CAGTGCAGAATTACCAGGTGTG-3′ and R: 5′- GGTCTGCCCTTTCTCCCTTCT-3′).

### 2.17 Quantitative RT-PCR

Total RNA from muscle tissues was isolated using Trizol reagent (Sangon, China) and reverse-transcribed to complementary DNA (cDNA) with the PrimeScript RT Reagent Kit (TAKARA, Japan). Amplification of cDNAs was performed using TB Green® Premix Ex Taq™ II (TAKARA, Japan) on a QuantStudio™ 6 Flex Real-Time PCR System (Life Technologies). Relative mRNA (mRNA) expression was determined by normalization to *GAPDH*, applying the 2^−ΔΔCt^ method. Primer sequences are listed in Table S1.

### 2.18 Western blot analysis

Muscle tissues were homogenized in ice-cold RIPA lysis buffer (Beyotime, China), enriched with 1 % PMSF to inhibit protease activity. After centrifugation at 12,000g for 20 min at 4 °C, the supernatant was carefully collected. Protein samples were separated by 8–15 % SDS-PAGE and transferred onto PVDF membranes. Following a 2 h blocking step with 5 % skim milk, membranes were probed with primary antibodies overnight at 4 °C, and then with horseradish peroxidase-conjugated secondary antibodies for 1 h. Protein bands were detected using an enhanced chemiluminescence (ECL) detection system (Tanon, China), and band intensities were quantified using ImageJ software (NIH, USA).

### 2.19 Statistical analysis

Data were analyzed utilizing GraphPad Prism software, version 8.0, and presented as mean ± standard deviation (mean ± S.D.). Comparisons between two groups were performed using the independent samples *t*-test, while multiple group comparisons were assessed via one-way analysis of variance (ANOVA) followed by Dunnett's multiple comparison test. Statistical significance was determined at the threshold of p < 0.05.

## 3 Results

### 3.1 Exercise attenuates skeletal muscle weakness in OVX mice

To investigate the effects of estrogen deficiency and exercise on skeletal muscle function, we established a mouse model of ovariectomy (*See Data S1 for details*) and subjected these mice to an 8-week moderate-intensity treadmill exercise program. While food intake remained comparable across all groups (*See* Fig. S1A *for details*), OVX mice exhibited a more rapid increase in body weight compared to sham-operated controls (*See* Fig. S1B *for details*). Exercise training effectively reduced body weight in OVX mice. Further analysis using small animal MRI revealed a significant increase in body fat percentage and a decrease in lean mass percentage in OVX mice (*See* Fig. S1C*, D for details*). Conversely, exercise training significantly reduced body fat content and increased relative lean mass.

Although absolute muscle weight was comparable among groups, relative muscle weight was significantly decreased in OVX mice (*See* Table S2 *for details*). Exercise training effectively increased relative muscle mass. Functional assessments, including grip strength and grip endurance, revealed a significant reduction in relative grip strength in OVX mice, indicating muscle weakness (Fig. 1A). Exercise training significantly improved grip strength. Additionally, OVX mice exhibited decreased grip endurance in both the inverted grid test and rotarod test, which was partially restored by exercise training (Fig. 1B and C).

**Fig. 1:**
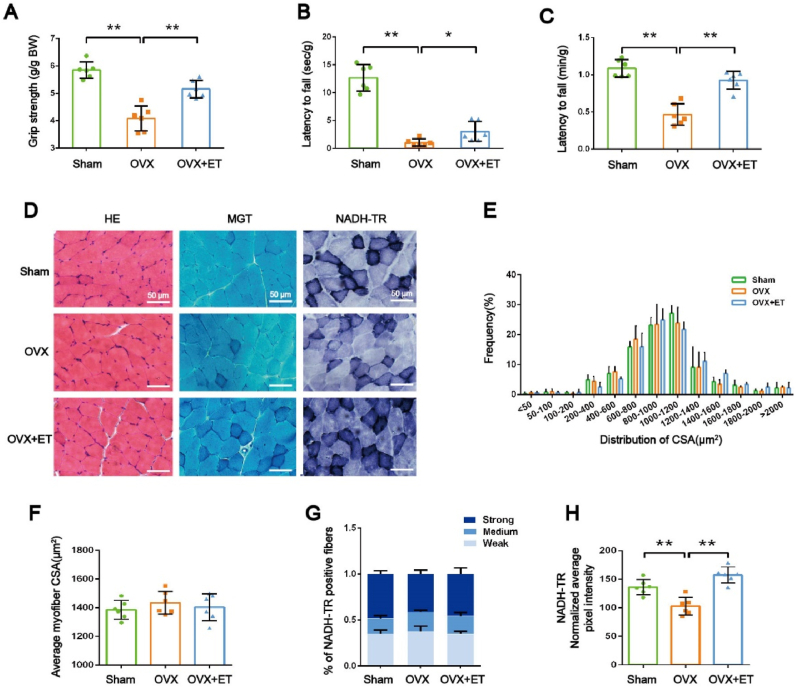
**Characterizations of the effects of exercise training on ovariectomized mice. *A,B,C,*** Changes in grip strength, inverted grid fatigability, and Rota-rod endurance of mice. ***D,*** Representative images of HE staining, MGT staining, and NADH-TR staining. Scale bar: 50 μm. ***E,*** Frequency of cross-sectional area (CSA) distribution for GAS muscles. ***F,*** Average CSA of GAS muscles. ***G,*** %NADH-TR positive fibers was quantified by calculating the percentage of weak, intermediate, or strong staining intensity of the whole muscle. ***H,*** NADH-TR activity was quantified by calculating the average of total pixel intensity. n = 3∼6 mice per group. ∗*P* < 0.05, ∗∗*P* < 0.01.

Given the established role of myosin regulatory light chain (RLC) phosphorylation in force generation, we assessed the phosphorylation status of RLC in the GAS muscle. OVX mice displayed a significant decrease in RLC phosphorylation, which was markedly increased by exercise training (See Fig. S2 for details). This suggests that exercise-induced improvements in muscle function may be partially mediated by enhanced RLC phosphorylation.

Histological analyses of the GAS muscle revealed no significant differences in muscle fiber size or collagen deposition among groups (Fig. 1D, E, F). However, OVX mice exhibited a significant decrease in mitochondrial content, as assessed by NADH-TR staining (Fig. 1D–G, H). Exercise training effectively increased mitochondrial content in OVX mice, suggesting that it may mitigate the negative effects of ovariectomy by promoting mitochondrial biogenesis.

### 3.2 Exercise training restores mitochondrial function and antioxidant capacity in skeletal muscle of ovariectomized mice

To further assess mitochondrial function, we performed high-resolution respirometry on permeabilized fibers from the GAS muscle. As shown in Fig. 2A and B, permeabilized fibers were sequentially exposed to different substrates and inhibitors to evaluate mitochondrial respiration. Compared to the sham group, OVX mice exhibited a significant decrease in various respiratory parameters, including state 4 respiration with malate and glutamate (complex I), state 3 respiration with ADP (complex I), state 3 respiration with succinate (complex I and II), state 3 respiration with rotenone (complex II), and state 3 respiration with ascorbate and TMPD (complex IV). Exercise training significantly reversed these decreases, indicating improved mitochondrial respiratory function. Additionally, we found that both ATP content and mitochondrial DNA (mtDNA) levels were significantly decreased in the GAS muscle of OVX mice, and these reductions were attenuated by exercise training (Fig. 2C and D). These findings suggest that exercise training may improve mitochondrial respiratory function by increasing mitochondrial content or quality.

**Fig. 2:**
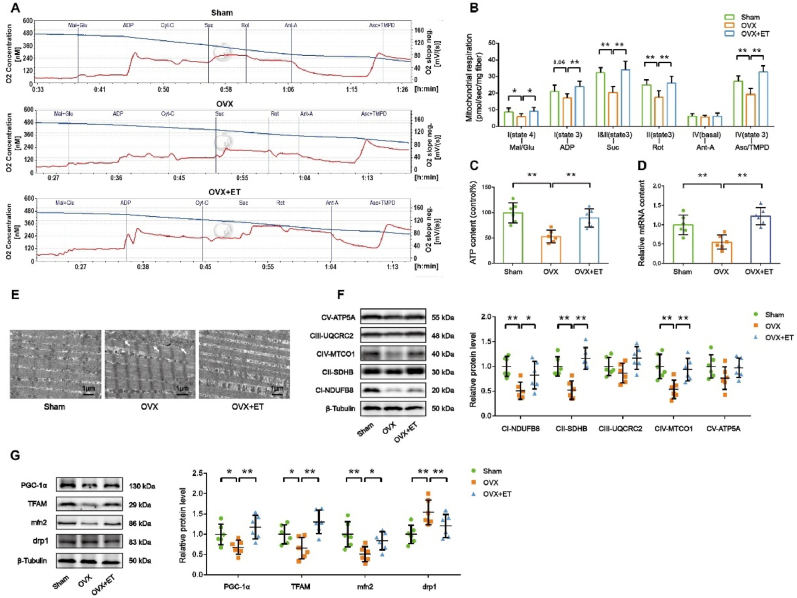
**Exercise training restores respiratory capacity, mitochondrial morphology, biogenesis, and dynamics in skeletal muscle of ovariectomized mice. *A,*** Oroboros O2k representative tracings of red gastrocnemius (RG). ***B,*** Mitochondrial-specific O2 flux in permeabilized fibers with Krebs cycle substrates and inhibitors. ***C,*** ATP content in GAS muscle. ***D,*** Mitochondrial DNA content evaluated by the ratio of a mitochondrial encoded gene (mt-Cytb) and a nuclear-encoded gene (Cycs). ***E,*** Representative electron micrograph of the intermyofibrillar area of the GAS muscle. Scale bar = 1 μm. ***F,*** Representative images of Western blot and semiquantitative analyses of the bands of OXPHOS proteins in GAS muscle. ***G,*** Representative images of Western blot and semiquantitative analyses of the bands of mitochondrial biogenesis and fusion/fission proteins in GAS muscle. n = 3∼6 mice per group. ∗*P* < 0.05, ∗∗*P* < 0.01.

Ultrastructural analysis revealed well-preserved muscle fiber morphology in all groups. However, ovariectomized (OVX) mice exhibited fewer and swollen mitochondria in the GAS muscle compared to sham-operated controls (Fig. 2E). Exercise training significantly increased mitochondrial number without morphological abnormalities in OVX mice.

Western blot analysis demonstrated decreased protein expression of mitochondrial respiratory chain complexes I, II, and IV in OVX mice (Fig. 2F). Exercise training significantly upregulated complexes I, II, III, IV, and V. Additionally, protein levels of PGC-1α and TFAM, key regulators of mitochondrial biogenesis, were reduced in OVX mice but were significantly increased by exercise training (Fig. 2G), suggesting enhanced mitochondrial biogenesis. To explore mitochondrial dynamics, we assessed fusion and fission proteins. Mfn2, a fusion protein, was downregulated, while Drp1, a fission protein, was upregulated in OVX mice. Exercise training reversed these effects, indicating a shift towards mitochondrial fusion (Fig. 2G).

**Fig. 3:**
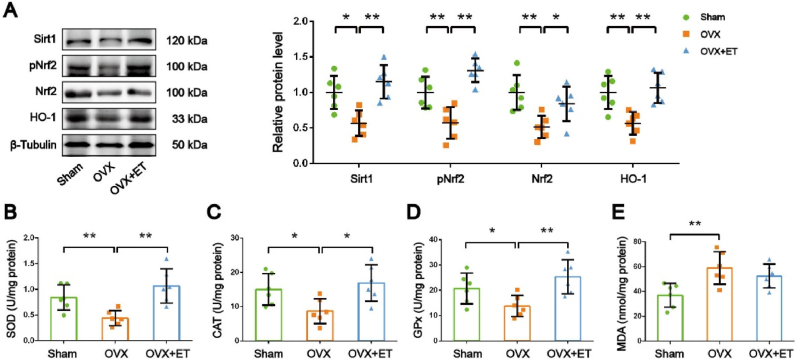
**Exercise training rescues antioxidant capacity in skeletal muscle of ovariectomized mice. *A,*** Representative images of Western blot and semiquantitative analyses of the bands of antioxidant signaling pathway proteins in GAS muscle. ***B, C, D, E,*** SOD, CAT and GPx activities and MDA content in mice GAS muscle. n = 6. ∗*P* < 0.05, ∗∗*P* < 0.01.

We also examined antioxidant signaling pathways. SIRT1, a metabolic stress sensor, was downregulated in OVX mice but was upregulated by exercise training. Similarly, pNrf2, Nrf2, and HO-1, key antioxidant proteins, were decreased in OVX mice and increased by exercise training (Fig. 3A). To assess antioxidant capacity, we measured superoxide dismutase (SOD), catalase (CAT), and glutathione peroxidase (GPx) activities. All three enzymes were significantly decreased in OVX mice but were increased by exercise training (Fig. 3B, C, D). Malondialdehyde (MDA), a marker of lipid peroxidation, was elevated in OVX mice, indicating increased oxidative stress (Fig. 3E). Exercise intervention showed a decreasing trend in MDA levels compared to OVX, but without statistical significance (Fig. 3E).

### 3.3 Exercise reverses muscle E2 levels and estrogen signaling in skeletal muscle of ovariectomized mice

**Fig. 4:**
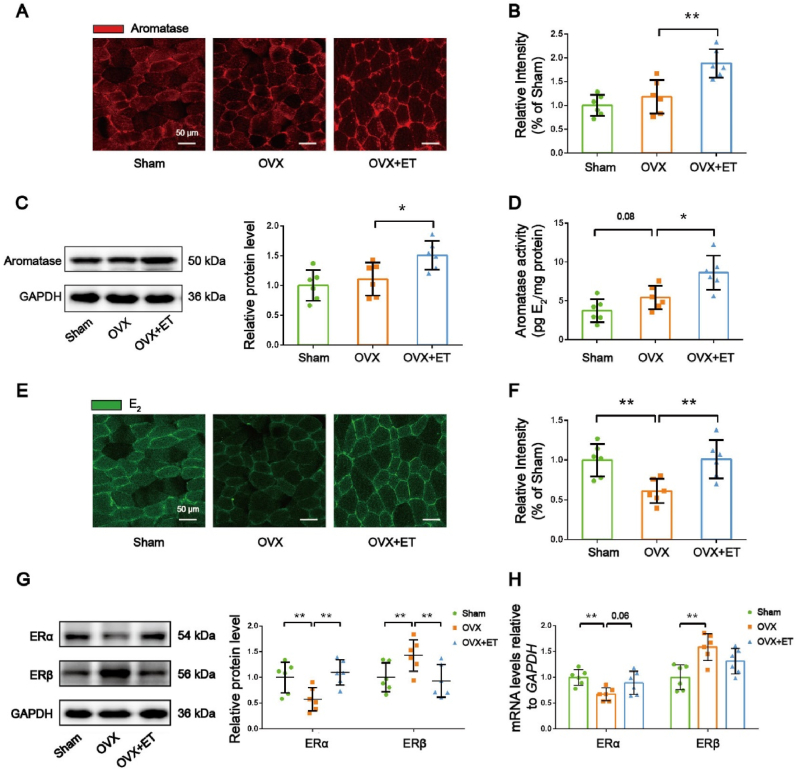
**Alterations in aromatase activity, E_2_ levels, and ER expression in GAS muscle. *A*,** Representative image of aromatase immunofluorescence assay. ***B***, Semiquantitative analyses of the fluorescence intensity of aromatase protein in GAS muscle. ***C***, Representative images of Western blot and semiquantitative analyses of the bands of aromatase protein in GAS muscle. ***D***, Aromatase activity analysis. ***E***, Representative image of estradiol immunofluorescence assay. ***F***, Semiquantitative analyses of the fluorescence intensity of estradiol in GAS muscle. ***G***, Representative images of Western blot and semiquantitative analyses of the bands of ERα and ERβ proteins in GAS muscle. ***H***, Quantitative analysis of the mRNA expression of ERα and ERβ in GAS muscle. n = 6 mice per group. ∗*P* < 0.05, ∗∗*P* < 0.01.

To examine the effects of exercise on myogenic estrogen synthesis, we performed immunofluorescence staining to assess aromatase expression in the GAS muscle. Results showed no significant difference in aromatase expression between the OVX and sham groups, while the OVX + ET group exhibited a significant increase in aromatase expression compared to the OVX group (Fig. 4A and B). Consistently, Western blot analysis revealed that exercise training significantly increased aromatase protein expression in the GAS muscle of OVX mice (Fig. 4C). Furthermore, aromatase activity assays demonstrated a trend towards increased aromatase activity in the GAS muscle of OVX mice compared to the sham group (*P=0.08, trending toward significance*), and exercise training significantly increased aromatase activity in the GAS muscle of OVX mice (Fig. 4D). Additionally, immunofluorescence staining revealed a significant decrease in E_2_ levels in the GAS muscle of OVX mice compared to the sham group, while exercise training significantly increased E_2_ levels in the GAS muscle of OVX mice (Fig. 4E and F). These findings suggest that ovariectomy significantly impairs skeletal muscle E_2_ levels, and exercise training increases intracellular E_2_ levels by upregulating aromatase expression and activity.

To further investigate whether exercise exerts its effects through estrogen receptors, we examined the expression levels of ERα and ERβ using qRT-PCR and Western blot analysis. Both qRT-PCR and Western blot analysis demonstrated that ovariectomy led to a downregulation of ERα expression in the GAS muscle (*qPCR results: OVX vs. Sham, P=0.06, trending toward significance*), and exercise training significantly reversed this decrease (Fig. 4G and H). Moreover, we found that ovariectomy increased the mRNA and protein expression of ERβ in the GAS muscle, while exercise training significantly downregulated ERβ protein expression in OVX mice without affecting ERβ mRNA expression (Fig. 4G and H).

### 3.4 Characterization of muscle-ARO-KO mice

**Fig. 5:**
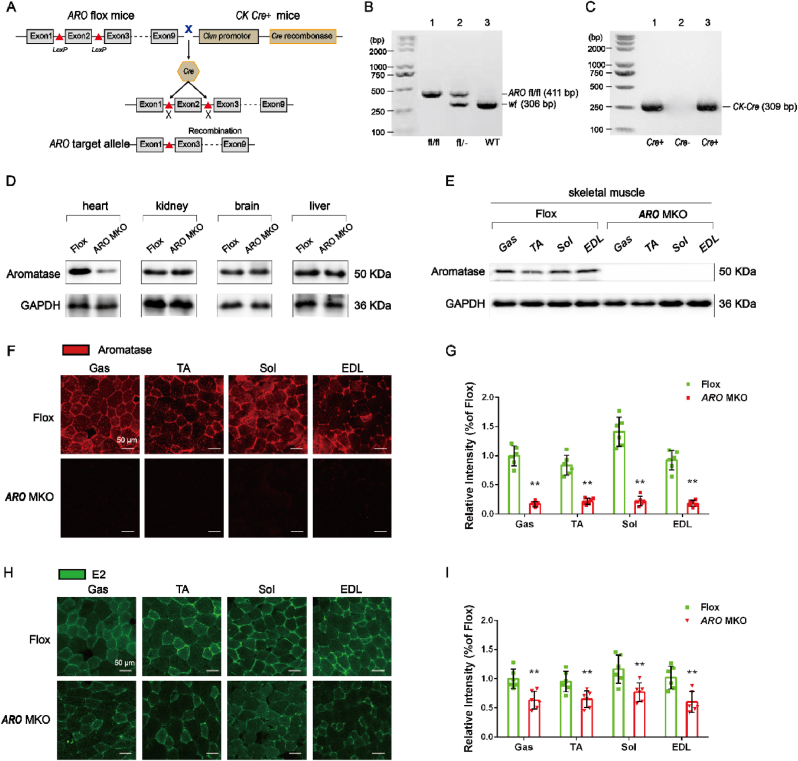
**Construction of muscle-specific *ARO* knockout mice. *A***, A chart described an organization-specific knockout strategy. ***B, C***, PCR detected *ARO* gene and Cre gene expression in mouse tail genomic DNA. ***D***, Western blot showed the expression of ARO in the heart, kidney, brain, and liver from *ARO*^fl/fl^ and *ARO*;Ckm-Cre female mice. ***E***, Western blot revealed the expression of ARO protein in the GAS, TA, SOL, and EDL muscles from *ARO*^fl/fl^ and *ARO*;Ckm-Cre female mice. ***F***, Immunofluorescence confirmation of aromatase expression (red) in GAS, TA, Sol, and EDL muscle. Scale bar: 50 μm. ***G***, Relative intensity of aromatase in GAS, TA, SOL, and EDL muscle was quantified following confocal analysis above. ***H***, Immunofluorescence confirmation of E_2_ level (green) in GAS, TA, SOL, and EDL muscle. Scale bar: 50 μm. ***I***, Relative quantification of E_2_ levels in GAS, TA, SOL, and EDL muscle. n = 6 mice per group. ∗*P* < 0.05, ∗∗*P* < 0.01 *vs*. Flox group.

To investigate the specific role of the *ARO* gene in skeletal muscle, we generated muscle-specific *ARO* knockout mice. The Cre-LoxP system was used to delete the second exon, a major functional domain, of the *ARO* gene specifically in muscle tissues, as illustrated in the schematic diagram (Fig. 5A). Genotyping of newborn mice confirmed the successful generation of floxed and Cre^+^ mice (Fig. 5B and C). We initially examined the expression of aromatase in various tissues, including skeletal muscle, heart, kidney, cerebral cortex, and liver. Western blot analysis revealed a significant decrease in aromatase expression in the heart of KO mice compared to WT mice, while there was no significant difference in aromatase expression in the kidney, brain, and liver between WT and KO mice (Fig. 5D). Subsequent cardiac function analysis did not reveal any significant differences between KO and WT mice (*See Data S2 for details*).

Compared to flox mice, *ARO* gene expression was absent in the GAS, TA, SOL, and EDL muscles of KO mice (Fig. 5E). Furthermore, immunofluorescence staining showed a significant decrease in red fluorescence signal representing aromatase in the GAS, TA, SOL, and EDL muscles of KO mice compared to WT mice (Fig. 5F and G). Similarly, green fluorescence signal representing E_2_ levels was also significantly decreased in the KO mice compared to WT mice (Fig. 5H and I).

Collectively, these results demonstrate the successful generation of muscle-specific *ARO* knockout mice.

### 3.5 Myogenic estrogen synthesis is dispensable for the exercise-induced preservation of mitochondrial function in skeletal muscle of ovariectomized mice

**Fig. 6:**
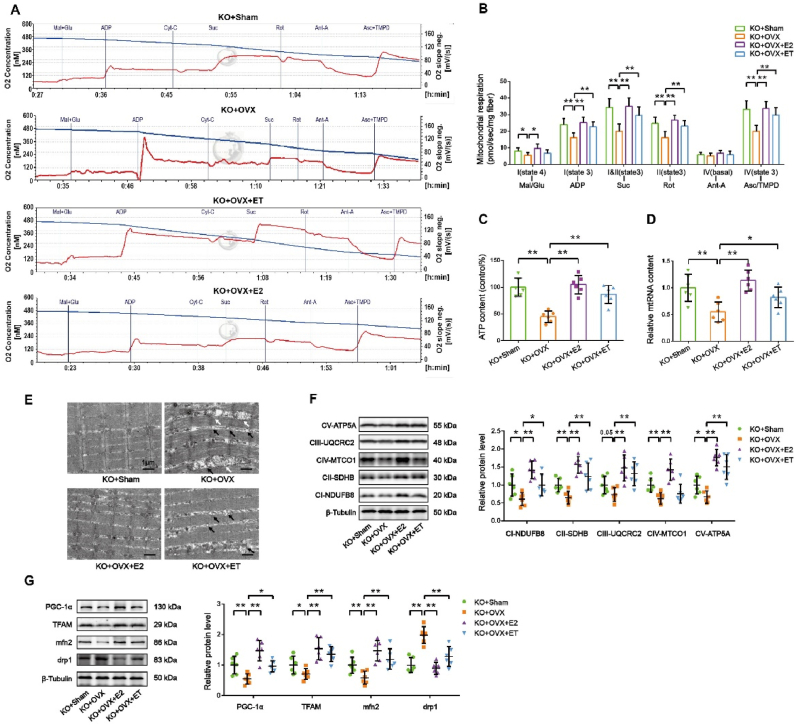
**Exercise training or E_2_ supplementation restores respiratory capacity, mitochondrial morphology, biogenesis, and dynamics in skeletal muscle of ovariectomized mice with *ARO* gene knockout. *A,*** Oroboros O2k representative tracings of red GAS (RG). ***B,*** Mitochondrial-specific O2 flux in permeabilized fibers with Krebs cycle substrates and inhibitors. ***C,*** ATP content in GAS muscle. ***D,*** Mitochondrial DNA content evaluated by the ratio of a mitochondrial encoded gene (mt-Cytb) and a nuclear-encoded gene (Cycs). ***E,*** Representative electron micrograph of the intermyofibrillar area of the GAS muscle. Scale bar = 1 μm. ***F,*** Representative images of Western blot and semiquantitative analyses of the bands of OXPHOS proteins in GAS muscle. ***G,*** Representative images of Western blot and semiquantitative analyses of the bands of mitochondrial biogenesis and fusion/fission proteins in GAS muscle. n = 3∼6 mice per group. ∗*P* < 0.05, ∗∗*P* < 0.01.

To investigate the impact of exercise or E_2_ supplementation on mitochondrial function in the skeletal muscle of ARO gene knockout mice following ovariectomy, we conducted high-resolution respirometry. As shown in Fig. 6A and B, compared to KO + Sham mice, KO + OVX mice exhibited a significant decrease in state 4 respiration with malate and glutamate (complex I), state 3 respiration with ADP (complex I), state 3 respiration with succinate (complex I and II), state 3 respiration with rotenone (complex II), and state 3 respiration with ascorbate and TMPD (complex IV). E_2_ supplementation significantly reversed these decreases. Furthermore, KO + OVX + ET mice showed a significant improvement in state 3 respiration with ADP (complex I), state 3 respiration with succinate (complex I and II), state 3 respiration with rotenone (complex II), and state 3 respiration with ascorbate and TMPD (complex IV) compared to KO + OVX mice. In addition, ATP content and mitochondrial DNA (mtDNA) levels were significantly decreased in the GAS muscle of KO + OVX mice, and both E_2_ supplementation and exercise training reversed these decreases (Fig. 6C and D).

Transmission electron microscopy revealed that while myofibril morphology was similar across all groups, KO + OVX mice exhibited swollen mitochondria with disrupted cristae or lack of cristae, and some mitochondria contained vacuolar structures (Fig. 6E). These mitochondrial abnormalities were attenuated in KO + OVX + E_2_ mice, suggesting a restorative effect of E_2_ supplementation. Similarly, exercise training was also observed to ameliorate mitochondrial abnormalities in KO + OVX mice, indicating that exercise might have a potential beneficial role in improving mitochondrial health following myogenic E_2_ deficiency.

Western blot analysis showed a significant decrease in the protein expression of complexes I, II, III, IV, and V in the GAS muscle of KO + OVX mice compared to KO + Sham mice (Fig. 6F*; CIII: KO + OVX vs. KO + Sham, P=0.05, trending toward significance*). E_2_ supplementation significantly increased the protein expression of these complexes, while exercise training increased the expression of complexes I, II, III, and V (Fig. 6F). Furthermore, we examined the expression of proteins involved in mitochondrial biogenesis and dynamics. As shown in Fig. 6G, the expression of PGC-1α, TFAM, and Mfn2 was significantly decreased, while the expression of Drp1 was significantly increased in the GAS muscle of KO + OVX mice compared to KO + Sham mice. E_2_ supplementation significantly reversed these changes. Similarly, exercise training partially restored the expression of PGC-1α, TFAM, and Mfn2, and suppressed the increased expression of Drp1 in KO + OVX mice.

**Fig. 7:**
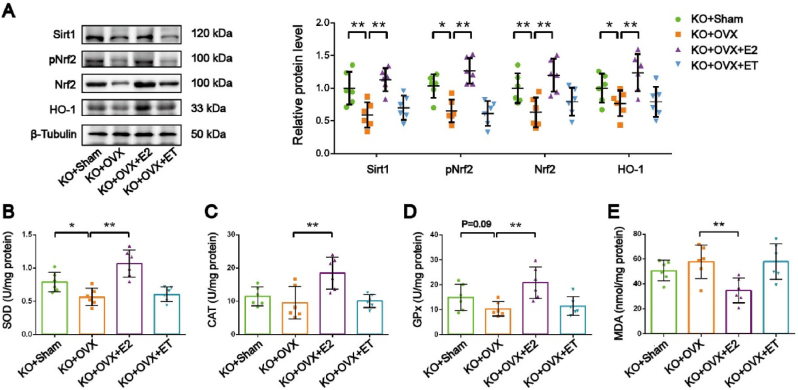
**E_2_ supplementation, but not exercise training, restored antioxidant systems in OVX mice with ARO knockout. *A,*** Representative images of Western blot and semiquantitative analyses of the bands of antioxidant signaling pathway proteins in GAS muscle. ***B, C, D, E,*** SOD, CAT and GPx activities and MDA content in mice GAS muscle. n = 6. ∗P < 0.05, ∗∗P < 0.01.

We next examined the expression of antioxidant signaling proteins in the GAS muscle. Compared to KO + Sham mice, KO + OVX mice exhibited a significant decrease in the protein expression levels of SIRT1, pNrf2, Nrf2, and HO-1, while E_2_ supplementation significantly upregulated the expression of these proteins (Fig. 7A). However, exercise training did not significantly affect the expression of SIRT1, pNrf2, Nrf2, and HO-1 in the GAS muscle of KO + OVX mice (Fig. 7A). Additionally, we examined the effects of exercise training or E_2_ treatment on antioxidant enzyme activities in the GAS muscle of OVX mice with *ARO* gene knockout. Compared to KO + Sham mice, KO + OVX mice showed decreased SOD (*P < 0.05*) and GPx (*P=0.08, trending toward significance*) activities, and E_2_ supplementation significantly increased the activities of SOD, CAT, and GPx (Fig. 7B, C, D); however, exercise training did not significantly affect the activities of SOD, CAT, and GPx in the GAS muscle of KO + OVX mice. Furthermore, E_2_ supplementation significantly reduced MDA levels, while exercise training had no significant effect on MDA content (Fig. 7E).

Collectively, these results indicate that myogenic E_2_ is not essential for exercise-induced mitochondrial protection, although the exercise-induced increase in myogenic E_2_ may provide additional benefits for antioxidant capacity.

## 4 Discussion

Mitochondrial health is crucial for maintaining optimal muscle performance in postmenopausal women. While exogenous estrogen supplementation can reverse estrogen deficiency-induced mitochondrial dysfunction, the role of exercise-induced myogenic estrogen (E_2_) production in ameliorating this condition remains unclear. Here, we showed that ovariectomy (OVX) in mice induced a muscle weakness phenotype, accompanied by impaired mitochondrial respiratory function and antioxidant capacity, which were partially reversed by exercise training. Moreover, exercise training upregulated aromatase activity and increased myogenic E_2_ production in the skeletal muscle of OVX mice. Notably, the protective effects of exercise on mitochondrial function persisted even after the specific knockout of the skeletal muscle aromatase gene, although the exercise-induced enhancement of antioxidant capacity was blocked. Our findings indicate that exercise improves mitochondrial function in the skeletal muscle of OVX mice through mechanisms independent of myogenic E_2_, while myogenic E_2_ plays a crucial role in enhancing skeletal muscle antioxidant capacity.

A significant and rapid decline in skeletal muscle function is observed postmenopause, closely associated with decreased ovarian estrogen levels [24,25]. Animal models have confirmed that ovariectomy (OVX)-induced estrogen deficiency leads to reduced skeletal muscle strength, an effect that can be reversed by estrogen supplementation [26,27]. Our study further explored the impact of estrogen deficiency on skeletal muscle in mice using the OVX model. Consistent with previous studies, OVX mice exhibited a significant increase in body fat mass without a significant change in food intake, suggesting metabolic disturbances caused by estrogen deficiency [28]. Concurrently, OVX mice showed an obvious decrease in grip strength and grip endurance, indicating muscle weakness. Although muscle mass and muscle fiber size remained largely unchanged, the phosphorylation level of myosin regulatory light chain (RLC) was significantly decreased, aligning with previous findings [26,29]. RLC phosphorylation is crucial for muscle contraction, and its reduction suggests impaired muscle contractile function [30,31]. Notably, compared to our 18-week OVX model, Yuriko et al.'s 24-week OVX study revealed a more pronounced atrophy of skeletal muscle CSA, suggesting that prolonged estrogen deficiency may contribute to more severe muscle wasting [32]. Exercise training effectively improved the phenotype of OVX mice, including reducing body fat mass, enhancing grip strength and endurance, and restoring RLC phosphorylation levels. These findings suggest that exercise can serve as a potent intervention to ameliorate muscle weakness induced by estrogen deficiency.

Typically, a decline in muscle strength is accompanied by a reduction in muscle mass. However, during aging, the loss of muscle strength may precede the loss of muscle mass, potentially due to multiple factors such as neuromuscular junction function, fiber type, and muscle composition [33]. In this study, we observed a decrease in NADH-TR staining intensity in the gastrocnemius muscle of OVX mice, suggesting potential mitochondrial impairment [34]. Previous research has documented the negative consequences of estrogen deficiency or impaired estrogen signaling on skeletal muscle mitochondrial bioenergetics, characterized by impaired mitochondrial respiration and reduced ATP content [10,11]. Our findings are in line with this, as we observed impaired function of mitochondrial complexes I, II, and IV in skeletal muscle of OVX mice, leading to significantly reduced respiratory rates. Exercise training, as expected, substantially ameliorated mitochondrial respiratory dysfunction in the skeletal muscle of ovariectomized mice, as evidenced by increased mtDNA copy number, oxidative phosphorylation protein expression, and ATP content.

Mitochondrial function and integrity are critically dependent on a complex quality control system involving biogenesis and dynamics (fission/fusion). PGC-1α is a key transcriptional co-activator regulating mitochondrial biogenesis by sensing cellular energy status and various stress signals, subsequently activating transcription factor A (TFAM) to promote mitochondrial DNA transcription, replication, and protein expression [35]. We found that OVX mice exhibited decreased expression of PGC-1α and TFAM proteins in the gastrocnemius muscle, indicating impaired mitochondrial biogenesis. Furthermore, we observed a downregulation of the fusion protein Mfn2 and an upregulation of the fission protein Drp1, suggesting decreased fusion and enhanced fission. This finding is in contrast to a previous study by Park et al., reporting decreased fusion and fission in the TA muscle of OVX mice [36]. The decline in estrogen levels can trigger a cascade of complex changes, and differences in muscle contractile properties and metabolic phenotypes may contribute to these discrepancies. Exercise training effectively restored the expression of these key regulatory proteins, preventing the development of abnormal mitochondrial morphology and preserving mitochondrial integrity. Consequently, the salutary effects of exercise on skeletal muscle in OVX mice appear to be substantially mediated by the preservation of mitochondrial function and integrity.

Considering the positive effects of exogenous E_2_ supplementation on skeletal muscle in ovariectomized (OVX) mice and the exercise-induced increase in myogenic E_2_, we generated a novel mouse model with muscle-specific *ARO* knockout to further investigate the role of exercise-induced myogenic E_2_ in mitochondrial function. While systemic aromatase deficiency has been shown to reduce muscle contractile function [37], our muscle-specific knockout mice exhibited preserved grip strength (*See Data S3 for details*), suggesting potential compensation by ovarian-derived E_2_ [38]. Our findings demonstrated that exogenous E_2_ supplementation effectively reversed estrogen deficiency-induced mitochondrial dysfunction, as evidenced by improved mitochondrial respiration, ATP production, and the expression of related proteins. Notably, exercise training restored mitochondrial function in KO + OVX mice independent of myogenic E_2_ production, indicating the existence of estrogen-independent compensatory pathways. Our data revealed exercise-mediated upregulation of PGC-1α and SIRT1, potentially driven by AMPK signaling, the primary energy sensor initiating skeletal muscle adaptation [39,40]. The AMP-activated protein kinase (AMPK) and NAD + -dependent deacetylase sirtuin 1 (SIRT1) co-regulate PGC-1α through phosphorylation and deacetylation respectively, coordinating mitochondrial biogenesis [41]. Functionally, AMPK may serve as the primordial trigger for exercise-induced adaptations in skeletal muscle and that activation of SIRT1 and its downstream signaling pathways [39,42]. Collectively, the AMPK-SIRT1-PGC1α axis may serve as a central compensatory pathway underpinning exercise-induced mitochondrial remodeling in the absence of estrogen. Future studies could explore pharmacological potentiation of this axis combined with exercise to optimize mitochondrial outcomes in estrogen-deficient models.

While our study showed that the protective effects of exercise on mitochondria are not solely dependent on myogenic E_2_, this factor may still significantly enhance skeletal muscle antioxidant capacity by activating the SIRT1/Nrf2 signaling pathway. SIRT1 can activate a series of antioxidant enzymes, such as HO-1, SOD, CAT, and GPx, by regulating the nuclear translocation of phosphorylated Nrf2, thereby exerting significant antioxidant effects [43,44]. Data from the wildtype mice model demonstrated that exercise training can activate the SIRT1/Nrf2 pathway in the gastrocnemius muscle of OVX mice, significantly increasing the expression and activity of antioxidant-related enzymes. However, the 8-week exercise training failed to significantly reduce skeletal muscle MDA levels in OVX mice. While exercise has been shown to significantly lower MDA in healthy individuals, its capacity to mitigate lipid peroxidation appears attenuated in pathological conditions such as hypercholesterolemia and diabetes [[45], [46], [47], [48]]. This suggests that the antioxidant response induced by an 8-week aerobic exercise regimen may not be sufficient to compensate for the oxidative stress generated in the skeletal muscle due to persistent estrogen deficiency. Our findings revealed that, in the absence of skeletal muscle aromatase, exogenous E_2_ supplementation, rather than exercise training, effectively increased the expression of antioxidant-related proteins, including SIRT1, pNrf2, Nrf2, and HO-1, in the gastrocnemius muscle of ovariectomized (OVX) mice. This finding is consistent with previous findings that exogenous E_2_ activates the SIRT1/Nrf2 pathway to inhibit oxidative stress and promote muscle repair [49]. Furthermore, we found that the absence of skeletal muscle aromatase disrupted the exercise-induced upregulation of antioxidant capacity in the gastrocnemius muscle of OVX mice, as evidenced by the increased activities of SOD, CAT, and GPx, and decreased MDA content, further confirming the crucial role of myogenic E_2_. Collectively, exercise-induced myogenic E_2_ generation may contribute to the promotion of antioxidant capacity in skeletal muscle.

Importantly, our findings demonstrate that exercise training in OVX mice enhances local E_2_ synthesis via aromatase activation while differentially modulating estrogen receptor subtypes (ERα↑, ERβ↓). The upregulated ERα, as a key mediator of estrogen action in skeletal muscle, may amplify E_2_-driven pathways critical for mitochondrial biogenesis and oxidative capacity [50,51]. This aligns with studies demonstrating ERα-specific knockout mice exhibit impaired contractility and fatigue resistance [52]. However, it is essential to recognize that ERβ also contributes to crucial functions in skeletal muscle, such as muscle cell regeneration, growth, and strength [53]. The downregulation of ERβ observed in our study may alleviate its potential antagonistic effects on ERα -mediated mitochondrial processes, particularly under estrogen -deficient conditions. It is important to note that the roles of ERα and ERβ can vary depending on the tissue and cellular context. For example, ERβ has been reported to enhance mitochondrial activity in non-muscle tissues (e.g., adipose and brain) [54,55], and its upregulation in adipose tissue during exercise contributes to improved metabolic homeostasis [56,57]. Future studies should elucidate how the hormonal milieu and cellular context determine the functional duality of ERβ and delineate the specific contributions of ERα and ERβ to exercise adaptation. This knowledge may inform targeted therapies for estrogen-deficient muscle dysfunction. While our data suggest that local E_2_ preferentially activates upregulated ERα while diminishing ERβ inhibition, the precise regulatory interplay between aromatase/E_2_ dynamics and ER subtype rebalancing remains to be clarified.

In conclusion, our study demonstrates that exercise can effectively preserve mitochondrial function in ovariectomized mice, even in the absence of myogenic estrogen synthesis. These findings underscore the critical role of exercise in maintaining skeletal muscle health and suggest that targeting mitochondrial function could be a promising therapeutic strategy for treating muscle weakness, particularly in conditions of estrogen deficiency. Future studies should elucidate whether exercise-induced mitochondrial protection involves AMPK-SIRT1-PGC-1α axis coordination with ERα/ERβ-specific contributions, and accelerating clinical translation through combinatorial regimens that integrate pharmacological activation of mitochondrial biogenesis with precision exercise prescription.

## CRediT authorship contribution statement

**Xu Tian:** Writing – original draft, Project administration, Data curation. **Yi Hu:** Project administration, Data curation. **Tao Li:** Project administration, Data curation, Conceptualization. **Fangfang Yu:** Project administration, Methodology, Data curation, Conceptualization. **Tingting Li:** Project administration, Data curation. **Xiangyang Tian:** Project administration, Methodology. **Yiwei Feng:** Supervision, Project administration, Data curation. **Qiuling Zhong:** Project administration, Data curation. **Yifan Meng:** Project administration, Data curation. **Wei Chen:** Validation, Formal analysis, Data curation. **Rengfei Shi:** Writing – review & editing, Supervision, Investigation, Funding acquisition.

## Funding

This work was supported by 10.13039/501100001809National Nature Science Foundation of China (No.32171136), 10.13039/100007219Natural Science Foundation of Shanghai (19ZR1452900), Key Laboratory of Exercise and Health Sciences (Shanghai University of Sport), Ministry of Education.

## Declaration of competing interest

The authors declare that there are no conflicts of interest in relation to the manuscript titled "Exercise-Induced Mitochondrial Protection in Skeletal Muscle of Ovariectomized Mice: A Myogenic E_2_ Synthesis-Independent Mechanism" submitted to ***Redox Biology***. We confirm that the results and interpretations reported in the manuscript are original and have not been plagiarized.

## Figures and Tables

**FigS1:**
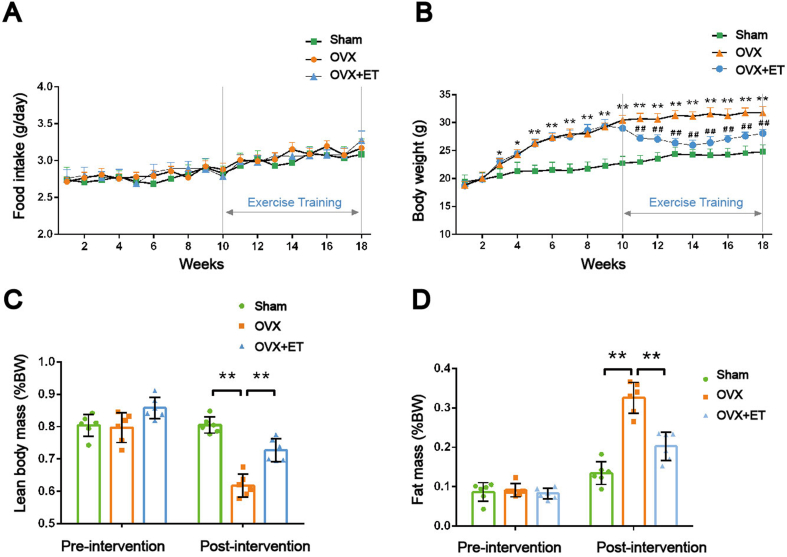


**FigS2:**
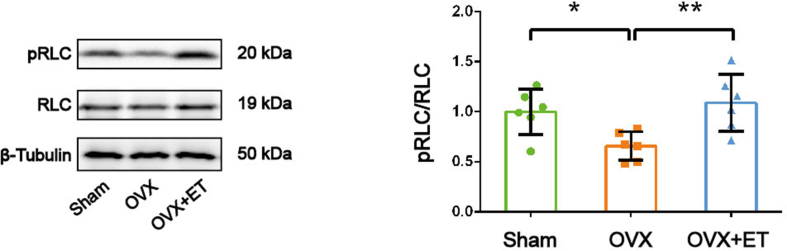


## Data Availability

Data will be made available on request.
