# Supplementary material for: Exercise-induced mitochondrial protection in skeletal muscle of ovariectomized mice: A myogenic E2 synthesis-independent mechanism
Source: Redox Biol. 2025 Jun 21;85:103735. doi: 10.1016/j.redox.2025.103735 (PMC12266561; doi:10.1016/j.redox.2025.103735)
Supplement: Multimedia component 1 [file mmc1.docx]

**Data S1. Supporting Information**

To confirm the success of the ovariectomy model, we evaluated vaginal cytology and uterine morphology. Sham-operated mice exhibited abundant cornified squamous epithelial cells in vaginal smears, indicating normal estrous cycles (Figure A). In contrast, ovariectomized mice showed predominantly leukocytes, consistent with estrogen deficiency (Figure A). Furthermore, uteri from sham-operated mice displayed a characteristic "Y" shape with well-developed horns, while those from OVX mice were significantly atrophied (Figure B). Serum estradiol (E_2_) levels were markedly decreased in OVX mice compared to sham-operated controls (Figure C). Collectively, these results demonstrate the successful establishment of the ovariectomy model.


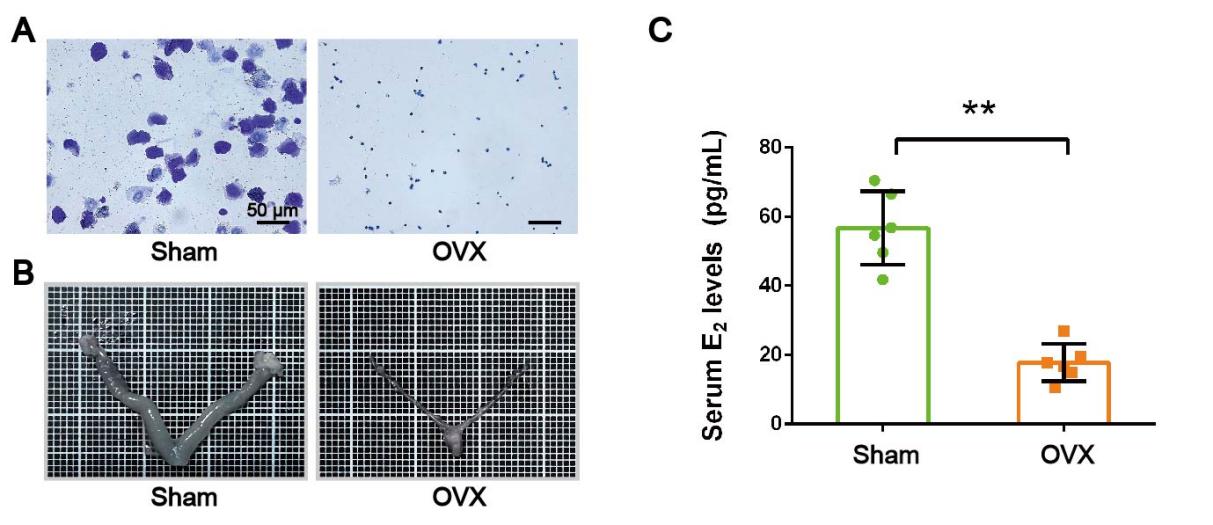


**Figure.** Changes in mice vaginal secretions, uterine morphology, and serum E_2_ levels after ovariectomized. Data are expressed as mean ± S.D. n = 6 mice per group. **P*<0.05, ***P*<0.01.
