# Supplementary material for: Exercise-induced mitochondrial protection in skeletal muscle of ovariectomized mice: A myogenic E2 synthesis-independent mechanism
Source: Redox Biol. 2025 Jun 21;85:103735. doi: 10.1016/j.redox.2025.103735 (PMC12266561; doi:10.1016/j.redox.2025.103735)
Supplement: Multimedia component 2 [file mmc2.docx]

**Data S2. Supporting Information**

Echocardiography was performed on 18-week-old mice using a Visual Sonics Vevo2100 ultrasound system to assess cardiac function. M-mode images of the left ventricle were obtained. Parameters such as beats per minute (Bpm), diastolic interventricular septum thickness (IVS, d), diastolic left ventricular posterior wall thickness (LVPW, d), diastolic left ventricle internal diameter (LVID,d), and ejection fraction (EF) were measured. No significant differences in cardiac function parameters were observed between aromatase knockout mice and aged matched floxed controls, in either male or female mice.

**Table. Effect of ARO KO on echocardiographic parameters determined in mice.**

|  | Female | | Male | |
| --- | --- | --- | --- | --- |
|  | WT | KO | WT | KO |
| Heart rate (bpm) | 474.3±19.3 | 471.6±17.2 | 470.7±16.5 | 467.9±16.8 |
| IVS,d (mm) | 0.72±0.03 | 0.74±0.02 | 0.76±0.03 | 0.79±0.02 |
| LVPW, d (mm) | 0.72±0.03 | 0.76±0.03 | 0.82±0.03 | 0.88±0.02 |
| LVID, d (mm) | 3.69±0.08 | 3.78±0.07 | 3.85±0.08 | 3.79±0.09 |
| EF (%) | 53.67±2.14 | 52.98±2.45 | 51.45±2.38 | 52.49±2.08 |

Data are expressed as mean ± SEM (n = 8-12 mice per group). Student's t-test, p>0.05 for all comparisons.
