# Supplementary material for: Exercise-induced mitochondrial protection in skeletal muscle of ovariectomized mice: A myogenic E2 synthesis-independent mechanism
Source: Redox Biol. 2025 Jun 21;85:103735. doi: 10.1016/j.redox.2025.103735 (PMC12266561; doi:10.1016/j.redox.2025.103735)
Supplement: Multimedia component 3 [file mmc3.docx]

**Data S3. Supporting Information**

After knocking out the ARO gene in muscle tissue, we did not observe any significant changes in grip strength ratio in either female or male mice. Additionally, ovariectomy led to a significant decrease in grip strength, but the absence of the ARO gene did not further exacerbate this decrease in female mice.


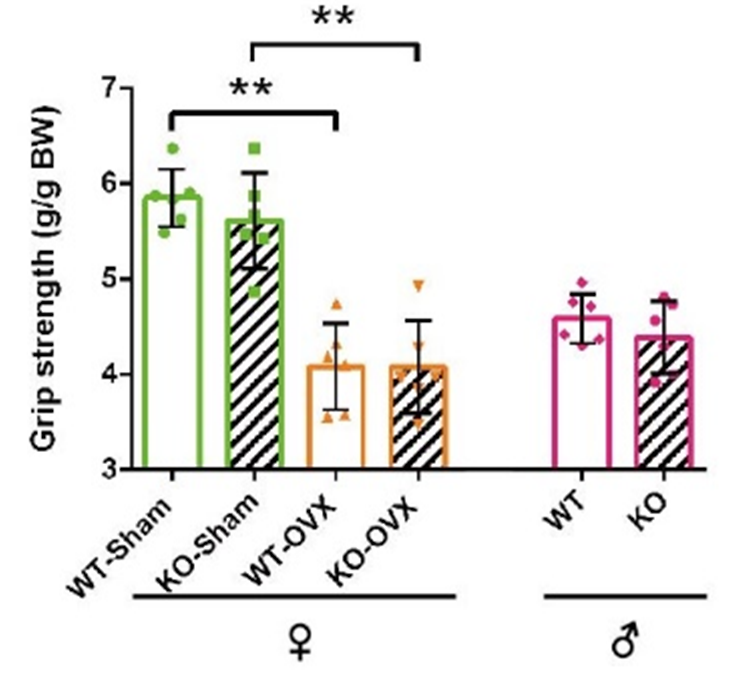


**Figure.** Effects of muscle *ARO* gene knockout on the grip strength of mice. Data are expressed as mean ± S.D. n = 6 mice per group. **P*<0.05, ***P*<0.01.
