# Supplementary material for: Exercise-induced mitochondrial protection in skeletal muscle of ovariectomized mice: A myogenic E2 synthesis-independent mechanism
Source: Redox Biol. 2025 Jun 21;85:103735. doi: 10.1016/j.redox.2025.103735 (PMC12266561; doi:10.1016/j.redox.2025.103735)
Supplement: Multimedia component 4 [file mmc4.docx]

**Table S1.** Primer sequence used for qRT-PCR.

| Gene name | Primer | Sequence(5'-3') |
| --- | --- | --- |
| *ERα* | Forward | CCTCCCGCCTTCTACAGGT |
|  | Reverse | CACACGGCACAGTAGCGAG |
| *ERβ* | Forward | CTGTGATGAACTACAGTGTTCCC |
|  | Reverse | CACATTTGGGCTTGCAGTCTG |
| *GAPDH* | Forward | AGGTCGGTGTGAACGGATTTG |
|  | Reverse | TGTAGACCATGTAGTTGAGGTCA |
