# Supplementary material for: Exercise-induced mitochondrial protection in skeletal muscle of ovariectomized mice: A myogenic E2 synthesis-independent mechanism
Source: Redox Biol. 2025 Jun 21;85:103735. doi: 10.1016/j.redox.2025.103735 (PMC12266561; doi:10.1016/j.redox.2025.103735)
Supplement: Multimedia component 5 [file mmc5.docx]

**Table S2.** Comparison of mice skeletal muscle weight.

| characteristics | Sham | OVX | OVX+ET |
| --- | --- | --- | --- |
| GAS weight (mg) | 153.13± 3.80 | 152.58± 3.15 | 153.08± 5.44 |
| TA weight (mg) | 59.29± 2.06 | 58.68± 2.79 | 58.81± 2.29 |
| EDL weight (mg) | 13.68± 0.95 | 13.37± 0.57 | 13.97± 0.91 |
| SOL weight (mg) | 9.96± 0.75 | 9.97± 0.55 | 10.37± 0.88 |
| Relative Gas weight (mg/g) | 6.18± 0.26 | 4.80± 0.25** | 5.45± 0.20## |
| Relative TA weight (mg/g) | 2.40± 0.10 | 1.85± 0.07** | 2.10± 0.13## |
| Relative EDL weight (mg/g) | 0.55± 0.06 | 0.42± 0.03** | 0.50± 0.04## |
| Relative Sol weight (mg/g) | 0.40± 0.04 | 0.31± 0.02** | 0.37± 0.03## |

GAS (Gastrocnemius), TA (Tibialis anterior), EDL (Extensor digitorum longus), SOL (Soleus); n = 6 mice per group. **P*<0.05, ***P*<0.01.
